# Supplementary material for: Efficiency of Simulation-Based Learning Using an ABC POCUS Protocol on a High-Fidelity Simulator
Source: Diagnostics (Basel). 2024 Jan 12;14(2):173. doi: 10.3390/diagnostics14020173 (PMC10814096; doi:10.3390/diagnostics14020173)
Supplement: Supplementary file 1 [file diagnostics-14-00173-s001.zip › Table S1.pdf]

Table S1. Evaluation criteria for the practical examination

Criteria	Score				
	1	2	3	4	5
1. Applied knowledge of ultrasound equipment (simulator)					
Familiarity with the equipment and its functions, correct selection of the probe, using buttons.	Unable to operate equipment		Operates the equipment with some experience		Familiar with operating the equipment
2. Image optimization					
Consistently ensuring optimal image quality by adjusting gain, depth, focus, frequency etc	Fails to optimize images		Competent image optimization but not done consistently		Consistently optimization of images
3. Systematic examination of the lungs<br>Consistently displaying systematic approach to the examination and presentation of relevant structures.	Unsystematic approach.		Systematic approach satisfactory.		Consistently displays a Systematic approach.
4. Systematic examination of the heart<br>Consistently displaying systematic approach to the examination and presentation of relevant structures.	Unsystematic approach.		Systematic approach satisfactory.		Consistently displays a Systematic approach.
5. Interpretation of images					
Recognition of image pattern and interpretation of findings	Unable to interpret any findings, unable to describe the images		Does not consistently interpret findings correctly, describes the images without giving a proper diagnosis.		Consistently interprets findings correctly, accurate ultrasound diagnosis.
6. Documentation of examination					
Documentation of imagies is consistent with the ultrasound scan images	Does not document any images		Documents most relevant images		Consistently documents relevant images
7. Duration of examination					

Making the examination in the given time	Unable to evaluate relevant structures in the given time	Able to evaluate some structures during the given time	Able to evaluate all structures in the proposed approach in the given time
