# Supplementary material for: Efficiency of Simulation-Based Learning Using an ABC POCUS Protocol on a High-Fidelity Simulator
Source: Diagnostics (Basel). 2024 Jan 12;14(2):173. doi: 10.3390/diagnostics14020173 (PMC10814096; doi:10.3390/diagnostics14020173)
Supplement: Supplementary file 1 [file diagnostics-14-00173-s001.zip › Table S2.pdf]

Table S2. Practical and theoretical assessment scores and final assessment diagnosis

Nr.	Evaluation criteria 1	2	3	4	5	6	7	Practical examination score	Pre-course MCQ score	Post-course MCQ score	Practical examination diagnosis	Recognition of diagnosis
1	3,3	1,0	4,0	2,0	3,7	4,0	3,7	3,1	73	91	Bilateral Interstitial syndrome	yes
2	4,0	2,3	5,0	4,0	4,7	4,7	5,0	4,2	82	94	Bilateral Interstitial syndrome	yes
3	4,0	2,7	3,3	4,3	4,3	4,3	5,0	4,0	89	90	Left moderate pneumothorax	yes
4	4,3	2,7	3,3	3,7	3,0	3,3	4,3	3,5	73	91	Inferior vena cava collapse/hipovolemia	yes
5	4,7	3,3	4,3	4,3	5,0	5,0	4,7	4,5	79	92	Cardiac tamponade	yes
6	4,7	2,7	3,3	3,0	3,3	3,3	4,7	3,6	85	94	Left lung interstitial syndrome	no
7	4,0	2,7	2,3	3,0	2,0	2,7	4,3	3,0	84	90	Right lung consolidation and small pleural effusion	no
8	4,7	3,3	4,7	4,7	5,0	5,0	5,0	4,6	85	84	Right ventricle failure/PE	yes
9	4,0	2,7	3,3	3,7	3,7	4,0	4,7	3,7	63	74	Inferior vena cava collapse/hipovolemia	yes
10	4,0	3,0	4,0	3,3	5,0	5,0	5,0	4,2	80	95	Right lung consolidation and small pleural effusion	yes
11	4,0	3,0	4,7	3,7	4,3	4,3	4,0	4,0	75	92	Bilateral Interstitial syndrome	yes
12	4,0	2,7	3,7	3,3	4,0	3,3	4,0	3,6	83	87	Right lung consolidation and small pleural effusion	yes
13	4,7	3,3	4,7	3,3	3,3	3,3	4,3	3,9	82	85	Reduced left ventricular ejection fraction	no
14	4,7	3,3	4,7	3,7	5,0	4,7	4,0	4,3	89	87	Left massive pneumothorax	yes
15	4,7	3,0	4,7	4,0	5,0	4,7	4,7	4,4	91	92	Cardiac tamponade	yes
16	5,0	3,0	4,3	4,0	5,0	5,0	4,7	4,4	76	88	Reduced left ventricular ejection fraction	yes
17	4,3	2,7	3,3	3,3	3,7	3,7	4,3	3,6	79	77	Cardiac tamponade	yes
18	3,0	2,3	3,7	2,7	3,7	3,7	3,7	3,2	73	83	Right massive pneumothorax	yes
19	4,7	3,0	4,3	3,7	4,7	4,7	4,7	4,2	57	72	Bilateral Interstitial syndrome	yes

20	3,7	3,0	4,3	3,3	4,3	4,3	4,3	3,9	86	91	Left massive pneumothorax	yes
21	3,3	2,7	4,3	3,0	4,0	4,0	4,0	3,6	61	87	Reduced left ventricular ejection fraction	no
22	4,7	3,0	4,3	3,3	4,3	3,3	4,3	3,9	65	87	left pleural effusion	yes
23	4,7	3,3	4,7	4,0	4,7	4,3	4,3	4,3	70	86	Right massive pneumothorax	yes
24	4,7	3,3	4,7	3,7	4,7	5,0	5,0	4,4	69	76	Right ventricle failure/PE	yes
25	4,0	2,7	3,7	3,7	3,0	3,0	3,7	3,4	82	94	Right lung consolidation and small pleural effusion	no
26	4,3	3,0	4,0	4,0	4,7	3,7	4,7	4,0	67	80	Cardiac tamponade	yes
27	5,0	3,3	4,7	4,3	4,7	3,7	4,7	4,3	60	83	Left massive pneumothorax	yes
28	4,0	2,7	4,7	4,3	4,0	4,0	4,7	4,0	67	83	Right lung consolidation and small pleural effusion	yes
29	4,7	3,3	4,3	4,7	5,0	3,3	5,0	4,3	86	89	Right lung consolidation and small pleural effusion	yes
30	4,3	3,0	4,7	4,7	4,7	3,3	4,7	4,2	96	87	left pleural effusion	yes
31	4,0	2,7	4,3	4,3	4,3	3,3	5,0	4,0	77	85	Bilateral Interstitial syndrome	yes
32	4,3	4,3	4,7	4,7	5,0	5,0	4,7	4,7	83	95	Right ventricle failure/PE	yes
33	5,0	3,3	5,0	4,7	5,0	5,0	5,0	4,7	88	83	Left massive pneumothorax	yes
34	5,0	3,0	4,7	4,0	5,0	4,7	5,0	4,5	87	93	Right ventricle failure/PE	yes
35	4,0	3,0	4,0	4,0	5,0	5,0	5,0	4,3	67	82	Reduced left ventricular ejection fraction	yes
36	4,0	3,3	4,7	4,7	5,0	4,7	4,7	4,4	83	90	Right lung consolidation and small pleural effusion	yes
37	4,0	3,3	4,7	4,0	4,7	3,0	5,0	4,1	70	82	Right ventricle failure/PE	yes
38	4,0	3,0	2,7	2,7	1,7	2,0	3,7	2,8	90	91	Left pleural effusion	no
